# Successful production of the potato antimicrobial peptide Snakin-1 in baculovirus-infected insect cells and development of specific antibodies

**DOI:** 10.1186/s12896-017-0401-2

**Published:** 2017-11-09

**Authors:** Natalia Inés Almasia, María Paula Molinari, Guillermo Andrés Maroniche, Vanesa Nahirñak, María Pilar Barrios Barón, Oscar Alberto Taboga, Cecilia Vazquez Rovere

**Affiliations:** 1 Instituto de Biotecnología, Centro de Investigación en Ciencias Veterinarias y Agronómicas, Centro Nacional de Investigaciones Agropecuarias, Instituto Nacional de Tecnología Agropecuaria, Repetto y De Los Reseros s/n, CP 1686 Hurlingham, Buenos Aires Argentina; 2 Consejo Nacional de Investigaciones Científicas y Técnicas, CONICET, Godoy Cruz 2290, C1425FQB Autonomous City of Buenos Aires, Argentina; 3 Facultad de Ciencias Agrarias, Universidad Nacional de Mar del Plata (UNMdP), km73,5 route 226, Balcarce, Buenos Aires Argentina; 4 LABINTEX-INTA, Agropolis Fondation, Montpellier, France

**Keywords:** Antimicrobial peptide, Cysteine-rich, Snakin-1, *Spodoptera frugiperda* Sf9 cells, Baculovirus expression system, GASA

## Abstract

**Background:**

Snakin-1 (StSN1) is a broad-spectrum antimicrobial cysteine-rich peptide isolated from *Solanum tuberosum*. Its biotechnological potential has been already recognized since it exhibits in vivo antifungal and antibacterial activity. Most attempts to produce StSN1, or homologous peptides, in a soluble native state using bacterial, yeast or synthetic expression systems have presented production bottlenecks such as insolubility, misfolding or low yields.

**Results:**

In this work, we successfully expressed a recombinant StSN1 (rSN1) *in Spodoptera frugiperda* (Sf9) insect cells by optimizing several of the parameters for its expression in the baculovirus expression system. The recombinant peptide lacking its putative signal peptide was soluble and was present in the nuclear fraction of infected Sf9 cells. An optimized purification procedure allowed the production of rSN1 that was used for immunization of mice, which gave rise to polyclonal antibodies that detect the native protein in tissue extracts of both agroinfiltrated plants and stable transgenic lines. Our results demonstrated that this system circumvents all the difficulties associated with recombinant antimicrobial peptides expression in other heterologous systems.

**Conclusions:**

The present study is the first report of a successful protocol to produce a soluble Snakin/GASA peptide in baculovirus-infected insect cells. Our work demonstrates that the nuclear localization of rSN1 in insect cells can be exploited for its large-scale production and subsequent generation of specific anti-rSN1 antibodies. We suggest the use of the baculovirus system for high-level expression of Snakin/GASA peptides, for biological assays, structural and functional analysis and antibody production, as an important step to both elucidate their accurate physiological role and to deepen the study of their biotechnological uses.

**Electronic supplementary material:**

The online version of this article (10.1186/s12896-017-0401-2) contains supplementary material, which is available to authorized users.

## Background

Phytopathogens are responsible for major agricultural losses, destroying crops and thus causing severe damage to the world economy. In addition to acute food shortages, the negative impact to production caused by bacteria, fungi, viruses and nematodes seriously undermines food security, which can result in malnutrition, migration and the death of humans and livestock. Antimicrobial peptides (AMPs) are present in diverse organisms such as insects, mammals, amphibians, fish, birds and plants, and have an important action in defense against pathogens. AMPs selectively target the DNA, RNA and proteins of pathogens providing a rich source of lead compounds for the discovery of promising novel antibiotics [1, 2]. In 1999, Segura et al. isolated an AMP from potato tubers (*Solanum tuberosum)* which was called Snakin −1 (StSN1). StSN1 exerts strong antimicrobial activity against phytopathogens [3–6] and animal pathogens [7]. In 2008, our group showed that transgenic potato plants overexpressing *StSN1* gene are tolerant to diseases caused by pathogens of major commercial importance [8]. We also demonstrated that this peptide participates in plant developmental processes and that is involved in redox homeostasis and hormone crosstalk [9–11]. Recently, we showed that in cv Kennebec potato Snakin/GASA (Giberellic Acid Stimulated in Arabidopsis) family consists of at least 18 members [12]. StSN1 is a cysteine-rich basic peptide of 63-amino acids (6922 Da) whose 3D structure is composed of two long α-helixes with six disulfide bonds, which are one of the major forces responsible for holding proteins in their respective conformations [13, 14]. However, information about biochemical characteristics, structural stability or even the mechanism of action of StSN1 is scarce and these studies require a convenient system for the high-level expression of the peptide.

The expression of cysteine-rich AMPs in *Escherichia coli* is a significant challenge because the formation of disulfide bonds in the recombinant protein is inefficient and leads to incorrect folding [15]. Several attempts to express StSN1 in *E. coli* have been made. The N-terminal tagging of the peptide with a *pel*B leader sequence, which enables its secretion into the periplasm, resulted in the formation of insoluble inclusion bodies (IB) containing a misfolded and biologically inactive peptide. Thus, StSN1 had to be purified, solubilized and refolded [6]. As an alternative approach, Kuddus et al. [16] expressed StSN1 expression via enhancing the formation of insoluble inclusion bodies in *E. coli* cells. Other strategies involving the fusion of StSN1 to the protein Glutathione S-transferase (GST) led to strong expression of the recombinant protein, but also in the form of IB [17, 18]. Meiyalaghan et al. [19] expressed StSN1 as a fusion to thioredoxin and obtained adequate quantities of soluble protein. However, the authors did not report the separation from thioredoxin. This last process might be difficult. For example, Herbel et al. [20] expressed tomato Snakin-2 (SN2) in *E. coli*, but could not separate it from thioredoxin by treating with thrombin and enterokinase enzymes. Thus, all strategies based on prokaryotic expression systems failed to simply produce a recombinant StSN1 peptide in a free soluble and functional state.

Recently, Kuddus et al. [21] reported the first work on the expression and purification of a fully active recombinant StSN1 in *Pichia pastoris*. These researchers obtained large amounts of pure recombinant StSN1 (rSN1) that exhibited strong antimicrobial activity against several microorganisms but, surprisingly, yeasts *P. pastoris* were also sensitive to rSN1 [21]. Besides, the difficulty to disrupt cells owing to their thick and hard cell wall is an additional potential disadvantage of the yeast expression system [22].

Chemical synthesis has also been used to produce disulfide-rich StSN1 and StSN2 peptides in an unfolded state that requires oxidative refolding and purification techniques to recover the functionally active peptides. This synthesis involves a laborious work using a combination of solid-phase synthesis, native chemical ligation and a subsequent cysteine/cysteine mediated oxidative folding to form the six internal disulfide bonds [5]. In addition, Meiyalaghan et al. [19] reported that mature lyophilized commercially synthesized StSN1 could not be solubilized due to the structural nature of the peptides. These observations highlight the difficulty of working with Snakin/GASA peptides. In addition, production of recombinant proteins in plant tissues is an expensive and time-consuming procedure, typically yielding suboptimal quantities of proteins that may not be sufficient for biological assays [6].

Antibodies that selectively identify proteins of interest are required for different assays (ELISA, Western Blotting, immunoprecipitation). The first report on the production of specific antibodies against StSN1 peptide is quite recent. Rong et al. [17] produced polyclonal GST-SN1 antibodies in mouse to develop immunoblots for the characterization of transgenic wheat lines. Similarly, Meiyalaghan et al. [19] obtained antibodies against the fusion protein his6-thioredoxin-SN1. Western blot analysis showed that the antibodies obtained from rabbit sera specifically recognized the StSN1 peptide that had been expressed in a wheat germ cell-free expression system, but cross reactive bands were also evident [19]. Following a different approach, Spinas [23] commercially designed six primary antibodies to recognize grapevine SN1 but none of them were able to detect SN1 from grapevine or potato in infiltrated *Nicotiana benthamiana* leaves.

The baculovirus expression system is a convenient and versatile eukaryotic system for heterologous gene expression and has many advantages over prokaryotic and other eukaryotic systems such as disulfide bond formation, oligomerization and high-level yields. Since the crystal structure of StSN1 reveals a unique protein fold with six disulfide crosslinks [24], this expression system would be advantageous for the expression of toxic proteins while most likely maintaining the correct folding.

The aim of this work was to evaluate the baculovirus system for the optimization of Snakin expression and their large-scale recombinant production. The parameters for StSN1 expression in the baculovirus system were optimized and the recombinant protein was then injected into mice for antibody production. The present study is the first report on the successful expression and purification of a Snakin/GASA peptide in insect cells and its use for the generation of specific anti-rSN1 antibodies.

## Results

### Expression vector construction for tagged Snakin-1 protein

**Fig. 1:**
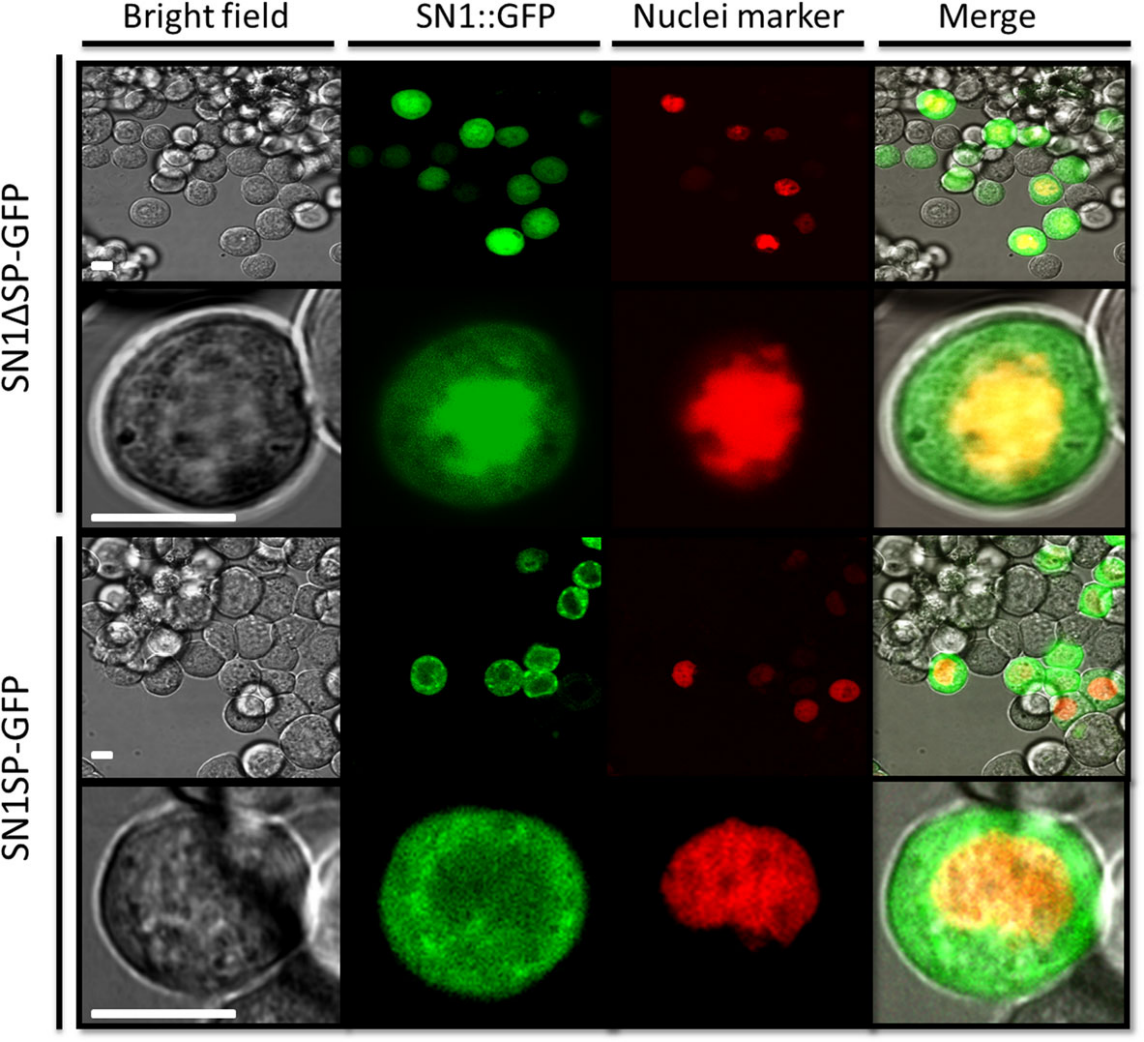
Subcellular localization of rSN1 in nuclei of Sf9 cells. StSN1 fused to GFP by the N-terminal end was transiently expressed in Sf9 cells and examined by live fluorescent imaging. Nucleus was visualized by mCherry-based fluorescent marker developed by Maroniche et al. [35]. In each case, images of the bright field (BF), signal of the fluorescent fusion with (SN1SP-GFP) or without the signal peptide (SN1ΔSP-GFP), nuclear marker and the merge of the images are shown. Scale bars: 10 μm

In order to express StSN1 peptide in *Spodoptera frugiperda* (Sf9) insect cells, we used the baculovirus Bac-to-Bac expression system. The first of the parameters that was considered for achieving an efficient protein expression method was the sequence of the peptide to be expressed. For this, we fused the complete and a truncated version of StSN1 lacking the putative signal peptide (SP) to Green Fluorescent Protein (GFP) at the N-terminal end in a vector suitable for insect cell expression (pIB-WG) and obtained SN1SP-GFP and SN1ΔSP-GFP fusion proteins, respectively. Transient expression assays were carried out in Sf9 cells and in vivo subcellular localization of the proteins was analyzed by confocal microscopy. As shown in Fig. 1, cells expressing SN1ΔSP-GFP yielded a stronger intensity within the nucleus and a weak homogeneous fluorescence throughout the cytoplasm. Coexpression of SN1ΔSP-GFP and a nuclear marker fused to mCherry showed high colocalization rates (0.86 Pearson’s coefficient). In turn, in cross-sections of cells transfected with SN1SP-GFP construct, green fluorescence was heterogeneously distributed in the cytoplasm and was almost excluded from the nucleus.

Hence, when the coding sequence for the mature StSN1 peptide was fused to GFP a conspicuous nuclear localization was observed. Considering that nuclei can be purified relatively easily, we used the sequence of the StSN1 lacking the signal peptide for further expression.

### Setting up for mass production of recombinant Snakin-1 protein in SF9 insect cells

To produce recombinant StSN1 protein (rSN1), we fused the sequence coding for the mature peptide downstream of the TEV protease cleavage sequence into pFastBac™HT B donor vector. After transformation of DH10Bac bacteria, six positive recombinant virus clones were identified by PCR. Simple small-scale isolation of the bacmid DNA was used for transfection of SF9 insect cells and several successive blind passages were conducted. Then, the viral titer and the multiplicity of infection (MOI) were calculated for four independent clones from two different rounds of amplification (second and third). The preliminary production of the recombinant protein was achieved by infection of fresh insect cell cultures with the resultant recombinant viruses. Total recombinant His-tagged protein was determined by Western blot in the crude extract. Thus, the optimal MOI that displayed a high expression of the rSN1 was estimated to be around 1.4–1.5.

**Fig. 2:**
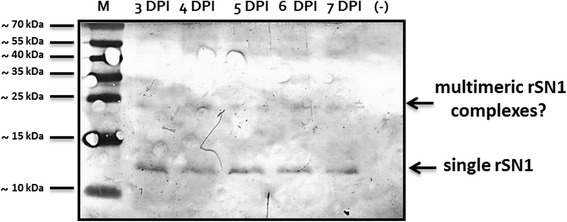
Production of recombinant protein in SF9 insect cells over time. Western blot analysis using an anti-HIS antibody (SDS-PAGE 10%) of crude extracts at different days after infection. (−) = mock-infected Sf9 cells extract. M: PageRuler Prestained Protein Ladder (Thermo Scientific). DPI: Days post infection

Then, insect cells were infected employing the optimal MOI with viral stock previously obtained. Cell samples were analyzed for recombinant protein production for several days to determine the optimal time for protein production. As shown in Fig. 2, rSN1 quantity, which was analyzed by Western blot, did not conspicuously decrease along post infection days, at least until 7 DPI. At 3 DPI, total extracts of Sf9 cells infected with the recombinant baculoviruses exhibited the most intense band with the expected electrophoretic mobility for tagged rSN1 (10,416 Da), which was absent in extracts of mock-infected Sf9 cells. Some additional bands of higher molecular weight were evident probably due to the formation of multimeric complexes.

**Fig. 3:**
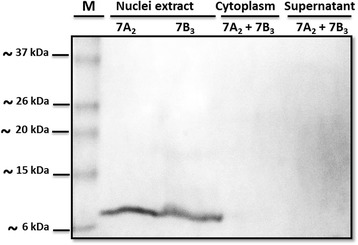
Production of recombinant Snakin-1 protein. Western Blot assay employing two independent recombinant virus (7A_2_ and 7B_3_ from the second and the third passage, respectively) and anti-His antibody. Concentrated supernatant, concentrated cytoplasm and nuclear fraction were evaluated. M: BenchMark Pre-Stained Protein Ladder. SDS-PAGE: 13.5%

To enrich the extracts with rSN1, we collected and fractionated cells into cytoplasms and nuclei using a non-ionic detergent. As it was expected, rSN1 localized mainly in the nuclear fraction, with little or no presence in the concentrated cytoplasm or supernatant fraction (Fig. 3). The apparent molecular weight of rSN1 was the estimated for the tagged protein and both clones employed (7A_2_ and 7B_3_) reached almost similar expression levels. Surprisingly, one single sharp band was observed, suggesting that multimeric complexes were disrupted throughout cell fractionation.

### Recombinant protein purification

**Fig. 4:**
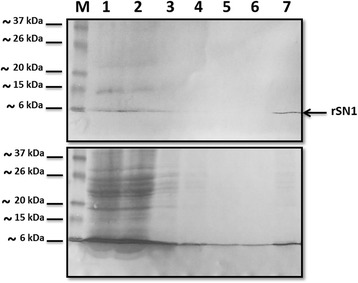
Purification steps of the rSN1 fusion protein. Upper panel: Western blot analysis using an anti-HIS antibody. Lane 1: Total nuclear lysate, Lane 2: Unbound proteins flowed through the column. Lanes 3–4: Proteins eluted from wash buffers of different stringent conditions tested (20 mM or 30 mM imidazole respectively). Lanes 5–6: Proteins eluted from acidic buffers of different pH 5.9 (lane 5) or pH 4.5 (lane 6). Lane 7: Proteins eluted (up to 6.3 mg/ml) with high concentrations of imidazole (500 mM). Lower panel: The nitrocellulose membrane stained with Ponceau dye for protein detection after the 10% SDS-PAGE analysis of representative purification steps of the rSN1. M: BenchMark Pre-Stained Protein Ladder. The arrow indicates the rSN1 peptide

After confirming the rSN1 accumulation in the nuclear fraction of insect cells, we selected clone 7A_2_ for further studies as it seemed to render slightly higher protein levels. After cell fractionation, disintegration of the nuclei was optimized. We rehearsed between two and five freeze-thaw cycles or sonication treatment of different intervals of time: pulses (1–5) seconds (30s, 60s). The optimal conditions for achieving a clarified homogenate were determined as 3 pulses of 30s of ultrasonic vibration to fragment the nuclear membranes and macromolecules. Then we performed purification of 6xHis-Tagged rSN1 from disrupted extracts using commercial Ni-NTA Spin Columns. We also fined tuned the three main stages of the purification: binding to Ni-NTA silica, washing and elution. Most of the non-tagged proteins flowed through (Fig. 4; Lane 2). Wash buffer of different stringent conditions were tested (15 mM, 20 mM or 30 mM imidazole) for removing residual contaminants and non-tagged proteins. Then, we tested different conditions to elute the rSN1 protein either by pH reduction (acidic values pH 4.5–5.9) or by competition with imidazole (500 mM) (Fig. 4). Finally, 100 mM EDTA was added since it strips nickel from the resin and none purified fusion protein was released from the column (data not shown). This confirms that the elution conditions were optimal.

We also tested the purification either under native or denaturing conditions, with or without the addition of protease inhibitors such as phenylmethylsulfonyl fluoride (PMSF), but none of these variants improved the results. Mechanical disruption of the nuclear fraction was crucial since yields (up to 25 mg of protein per 10^9^ cells) and purity varied depending on the viscosity of the initial lysate. Altogether, most of the tagged protein bound accurate to the silica resin under native conditions and elution was successful only with high concentrations of imidazole.

### Obtaining polyclonal serum against recombinant protein rSN1

**Fig. 5:**
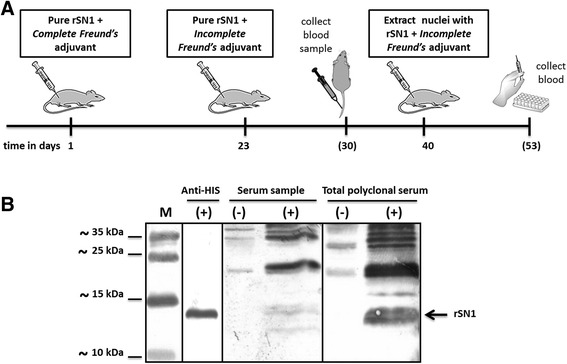
Scheme of inoculation and serum obtention. **a** Mice were immunized at Day 1, a booster dose was given at Day 23, a serum sample was taken at Day 30, an additional booster dose of incomplete Freund’s adjuvant (IFA) emulsion was given at Day 40 by employing extract nuclei expressing rSN1; and total serum was collected at Day 53. **b** Western blot analysis of extract nuclei of Sf9 cells either expressing rSN1 (+) or not (−). M: PageRuler Prestained Protein Ladder. Three different antibodies were used to reveal commercial anti-HIS, serum sample and total polyclonal serum (15% SDS-PAGE). The arrow indicates the rSN1 peptide

We then proceeded to produce anti-rSN1 antibodies. For this purpose, BALB/c mice were injected with a prime dose of pure rSN1 emulsified in complete Freund’s adjuvant and subsequently two boosters to the immunizing antigen were performed using incomplete Freund’s adjuvant (Fig. 5a). Serum samples were taken 13 days after the first booster dose, and antibody titers were tested by Western blot (serum sample, Fig. 5b). Finally, an additional booster dose was given employing total extract nuclei Sf9 cells expressing rSN1 and total serum was collected, by the sacrifice of the animal, 13 days after administration of this last booster dose. As shown in Fig. 5b, both polyclonal rSN1 antibody raised in the injected mouse and commercial anti-HIS antibody detected the protein rSN1 tagged with 6xHis. The observed band was more intense with the serum sample than with the commercial anti-His antibody, but less intense than with the total serum collected (total polyclonal serum) (Fig. 5b). The anti-rSN1 revealed some additional bands of higher molecular weight in the Western blot, since the analysis was performed with total sera and total extract nuclei of Sf9 cells. Thus, we selected total polyclonal serum for further assays to detect the protein StSN1.

### Evaluation of the effectiveness of anti-rSN1 antibody serum

**Fig. 6:**
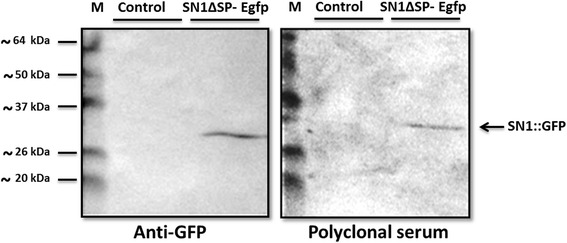
Detection of SN1::GFP expressed by agroinfiltration in *N. benthamiana* plants. SN1ΔSP-Egfp: Protein extract from agroinfiltrated *N. benthamiana* with a vector capable of expressing SN1::GFP (~34 kDa); Control: Protein extract of *N. benthamiana* agroinfiltrated with an empty vector; M: BenchMark Pre-Stained Protein Ladder. Western blot analysis revealed either with Anti GFP or the anti-rSN1 polyclonal serum is shown, 10% SDS-PAGE. The arrow indicates the SN1::GFP fusion protein

We then evaluated the presence of specific antibodies against StSN1 in the total polyclonal serum. For this purpose, we performed Western blot assays using protein extracts from agroinfiltrated *Nicotiana benthamiana* leaves expressing SN1::GFP. The polyclonal serum anti-rSN1 and the commercial anti-GFP antibody effectively detected the fusion protein (Fig. 6). Inmunoblot analysis showed that the mouse serum specifically recognized the SN1::GFP fusion protein transiently expressed in plant tissues, demonstrating that the serum contain specific antibodies against StSN1. Subsequently, we determined the serum titer by immunoblots assays. When purified rSN1 was employed as epitope, the titer was 1,4 × 10–^5^ but when total crude leaf protein was used, the established titer was 3,3 × 10–^3^. This result indicated that although the band was visible up to Dilution 1:300, the optimal dilution of the antibody for plant extract was 1:100 (Additional file 1: Figure S1). Specific antibodies developed in this work demonstrated to be a useful tool when anti-rSN1 serum successfully detected a more intense band in StSN1-overexpressing lines compared to non-transformed control plants (Additional file 2: Figure S2).

## Discussion

Nowadays, there is growing interest in AMPs because these compounds can be widely used in the fields of pharmaceutics, cosmetology, veterinary medicine, aquaculture, agricultural and food industries [21]. Elucidation of mechanisms of action and toxicity of AMPs may be the key to future therapeutic applications [25, 26]. In this context, the development of an efficient expression method is essential. Investigations on members of antimicrobial peptides Snakin/GASA family had highlighted the difficulty of working with these peptides [19] because they are toxic to bacteria and yeasts. Indeed, we could only recover one small colony after the transformation of *E. coli* DH5α with SN1-pFastBac and incubation for 2 days at low temperature, which illustrates rSN1 toxicity for prokaryotic cells even when expressed at low levels from cryptic promoters present in the plasmid. To circumvent the toxicity problem, we expressed StSN1 peptide using the baculovirus system since it presents numerous advantages for producing soluble recombinant proteins. As a result, we showed that baculovirus-infected insect cells can be used to produce high levels of soluble rSN1 peptide, indicating that it is indeed a suitable heterologous expression system for AMPs. In distinction to previous reports on prokaryotic and synthetic systems, and similarly to *Pichia pastoris* system, we have successfully developed a protocol to produce a large amount of soluble and native rSN1 peptide in baculovirus-infected cells.

We demonstrated that cells expressing SN1ΔSP-GFP yielded a strong fluorescence into the nucleus and also a weaker signal in the cytoplasm. Nonetheless, rSN1 not fused to GFP was totally recovered from the nuclear fraction of the infected insect cells. This can be explained by GFP interference, physiological alteration of the cells upon infection or different expression level. Although the presence of rSN1 in the nucleus can be expected because small proteins usually diffuse through the nuclear pore complexes [27], its exclusive nuclear localization suggests that it is retained inside this organelle. Using the prediction servers PROSITE (https://prosite.expasy.org/prosite.html) or cNLS Mapper (http://nls-mapper.iab.keio.ac.jp/cgi-bin/NLS_Mapper_form.cgi), no potential nuclear localization signal (NLSs) could be found. The interaction of rSN1 with nuclear factors, or with DNA, is a plausible and intriguing alternative explanation. A similar localization has been observed for vankyrin proteins, which lack apparent nuclear import and export signals, suggesting that different cellular factors interact with them [28]. In plant cells, we also showed that SN1ΔSP-Egfp is presented in the cell nucleus [10]. Finally, in insect cells SN1SP-GFP showed a heterogeneous cytoplasmic distribution, indicating that somehow the signal peptide (SP) of StSN1 is functional.

In the current work, we performed optimization of some parameters such as MOI, post infection days to collect, sonication, native or denaturing conditions, among others. We conclude that a crucial purification step was the homogenization of the nuclear fraction; the nuclei had to be sonicated until obtaining a low viscosity clarified homogenate to recover the bulk of the produced protein. Our study demonstrates, for the first time, that the nuclear localization of rSN1 in insect cells can be used for the large-scale production of this peptide, which is important for further structural and functional analysis. Western blot analysis of total insect cells extracts revealed a band of the expected size for rSN1 and some additional bands of higher molecular weight, presumably formed because StSN1 self-interacts in vivo as we previously showed by bimolecular fluorescence complementation assays [10]. After purification, StSN1 antibodies were raised against the recombinant protein. Western blot analysis performed with extracts from agroinfiltrated leaves exhibited only one sharp band, demonstrating that the anti-rSN1 serum was able to detect specifically StSN1 peptide expressed in plants. Similarly, Meiyalaghan et al. [19] showed in a previous work that antibodies obtained from rabbit sera were able to recognize StSN1 expressed in a wheat germ cell-free expression system; however, they also detected cross reactive bands in Western blot analysis. Here, the anti-rSN1 polyclonal serum allowed us to confirm that previously obtained StSN1 transgenic potatoes, with promising biotechnological application, [8] overexpress almost equal amounts of StSN1. Surprisingly, the molecular weight of the detected protein was higher than expected. This may be due to StSN1 in vivo self-interaction, or to the attachment/interaction with other molecules, as it was already reported for Snakin proteins with a Pro-rich protein (PRP) [29], with the sucrose transporter StSUT1 [30] or binding metal atoms [31]. The antibodies obtained in this work will also be useful for future studies on biochemical and structural properties of StSN1 peptide.

Here, the purification of rSN1 was aided by the presence of a polyhistidine affinity tag. In a previous study in a prokaryotic system, StSN1 was engineered with the same tag but the attempts to purify the resulting protein, after IB solubilization and refolding, failed. Since the presence of the tag was confirmed, they hypothesized that their inability to affinity-purify the target protein could be due to some conformational alterations that render the tag inaccessible for binding to the resin [6]. Since baculovirus system is able to produce eukaryotic post-translational modifications and since we succeeded at purifying the rSN1 using a resin column, rSN1 disulfide bonds are likely correctly formed stabilizing the globular structure, a conformation that would favor the accessibility of the tag. Besides, the relatively small size and charge of this polyhistidine tag ensure that the protein activity is rarely affected [32]. In addittion, several investigations proved that the presence of this tag had no impact on protein activity [33] but the antimicrobial activity of rSN1 against different microorganisms is still under investigation. In a recent work, Kuddus et al. [21] demonstrated that StSN1 functions as a cationic antimicrobial peptide, exerting its antimicrobial effects via perturbation of the cell membrane. Similarly, StSN2 shows a non-specific pore-forming effect in all tested membranes, so it was proposed as a preservative agent to protect food, pharmaceuticals or cosmetics from decomposition by microbes [34]. Since both Snakin peptides exhibit a similar spectrum of activity against microorganisms [3], it remains to be studied whether rSN1 could be useful as a preservative as well.

## Conclusions

The baculovirus expression system showed to be a convenient and versatile eukaryotic system for heterologous gene expression of Snakin/GASA peptides. Here, we optimized the parameters for StSN1 recombinant expression and then produced antibodies by injecting the protein into mice. The present study is the first report on the successful expression of recombinant StSN1 in insect cells and its use for the generation of anti-rSN1 antibodies. Since StSN1 peptide exhibit a broad spectrum of activity against important microorganisms, it could be used in agricultural biotechnology and as preservatives as well. Finally, we suggest the use of the baculovirus system for high-level expression of Snakin/GASA peptides, as an important step to both elucidate their accurate physiological role and to deepen the study of their biotechnological uses.

## Methods

### Plasmids

For this study the open reading frame for Snakin-1 (StSN1, GenBank: EF206292.1) was amplified by PCR employing potato *Solanum tuberosum* cDNA as template. The primers used for this amplification were as follow: Up: 5’GGATCCGGTTCAAATTTTTGTG3’and Low: 5’AATACAGGATCCTCAAGGGCATTTAGACTTGCC3’, the sequence of BamHI recognition site was added in both primers. The 210 bp amplified sequence was cloned into pGEMT (Promega, USA), sequenced for authentic amplification through commercial sequencing facilities and the sequence of interest (coding for the 63 aminoacid mature peptide) was released with BamHI. The resulting sequence were cloned in the baculovirus transfer vector pFastBac 1 (pFB1; Invitrogen), as a BamHI - BamHI fragment. Surprisingly, positive colonies were the little ones after growing 2 days at room temperature. PCR analysis, of the flanking insertion region and the gene of interest, and restriction assays confirmed the introduction of the SN1 gene into four independent vector clones in the correct sense.

For the subcellular localization assays, the collection of pCR8/GW/TOPO (Invitrogen, USA) entry vectors containing SN1 coding sequences with or without their signal peptide (SP) [10] were used. For live imaging in insect cells, the Gateway destination vector pIB-WG developed by Maroniche et al. [35] was used. Amino and carboxi-terminal GFP fusion proteins were obtained using the LR Clonase II enzyme mix (Invitrogen, USA) according to the manufacturer’s protocol. Nuclei of insect cells were tracked using a mCherry-based fluorescent marker [35].

### Fluorescence live imaging

For the subcellular localization assays a protocol described by Maroniche et al. [35] was used. Briefly, Sf9 cells were seeded into 35 mm dishes using Sf900II Serum free media medium (Invitrogen, USA) supplemented with 2% fetal bovine serum (Invitrogen, USA), and incubated at 27 °C until 60–70% confluence. The cells were then transfected with 1 μg of each plasmid (SN1 with or without signal peptide and nucleus marker) using Cellfectin II transfection reagent (Invitrogen, USA) according to the manufacturer’s instructions, and incubated at 27 °C until used. At 48–72 h post-transfection, the culture medium was replaced with phosphate buffered saline (PBS, pH 6.2) and transfected cells were used for fluorescence imaging in a LeicaTCS-SP5 (Leica Microsystems GmbH, Wetzlar, Germany) spectral laser confocal microscope (Laboratorio Integral de Microscopía, CICVyA, INTA) using a 63 × objective (HCX PL APO CS 63.0 × 1.20 WATER UV). The 488 nm line of the Argon laser and the 543 nm line of the HeNe laser were employed for GFP and mCherry excitation, respectively. Scanning was performed in sequential mode to minimize signal bleed-through and fluorescence emission was detected in 498–540 nm for GFP and 610–670 nm for mCherry. The microscope power settings, detectors gain and scanning speed were adjusted to optimize contrast and resolution for each individual image. Co-localizations were analyzed by calculating the Pearson’s correlation coefficient with the co-localization module of the Leica LAS AF software.

### Cell lines and generation of recombinant baculoviruses using the BAC-to-BAC system

*Spodoptera frugiperda* Sf9 cells (IPLBSF21-AE clonal isolate 9) were grown in Grace’s insect cell medium supplemented with 10% fetal calf serum (FCS; Gibco-Invitrogen). Sf9 cells were grown in T175 flasks at 27 °C. Cells were seeded at 0.5 × 106 cells/ml and infected when the cell densities reached 3,5 × 10^7^ cells/ml. The first component of the system is a pFastBac vector, into which the StSN1 gene was cloned to create the recombinant donor plasmid. The *Escherichia coli* cell line DH10BAC, which carries the baculovirus genome cloned into a bacterial artificial chromosome (BAC), was used to introduce the StSN1 gene expression cassette using transposition methods according to the manufacturer’s protocol (Invitrogen), adding kanamycin (50 μg/ul); tetracyline (10 μg/ul), gentamicin (7 μg/ul); x-gal (100 μg/ul) and IPTG (40 μg/ul). PCR analysis of the bacmids DNA was performed to confirm the introduction of the StSN1 gene into the baculovirus employing M13F and M13R commercial primers, and four independent positive bacmids clones were selected.

### Recombinant baculovirus production

Sf9 cells (0.5 × 10^6^ cells in T175 cell culture flasks, Corning, Inc.) were transfected with bacmid DNA using Cellfectin reagent (Invitrogen) as previously described by Molinari et al. [36]. Four independent bacmid clones were transfected for StSN1 gene. All recombinant baculoviruses were propagated in Sf9 cells grown at 27 °C in TNM-FH medium (SIGMA) supplemented with 10% fetal bovine serum (FBS) and antibiotic-antimycotic solution (GIBCO). Infectious virus titers were calculated by end point dilution assay and converted to PFU ml^−1^ as described by O’Reilly et al. [37] and optimal multiplicity of infection (MOI) was also determined. Virus Stock titers were typically 10^8^ PFU/ml. Sf9 cells grown in 25 cm2 flasks were infected at 1.5 MOI with the recombinant baculovirus. From 3 to 7 days post infection (DPI), cells were harvested, clarified (5 min at 300 g), lysed by boiling in presence of 500 μl cracking buffer (50 mM Tris–HCl pH 6.8, 2% SDS, 0.01% bromo-phenol blue, 1% 2-mercaptoethanol, 10% glycerol) and proteins were resolved by SDS-PAGE. Recombinant proteins were detected by Western blot employing anti-HIS antibody., Sf9 cells grown in T175 flasks were resuspended in 10 ml de PBS (pH 6.2), shaked strongly and centrifuged (5 min at 300 g) to collect nuclei cells. Precipitated cells were resuspended in 4 ml de Lisis Buffer (TrisHCl pH 8 50 mM, NaCl 150 mM, NP40 1% and Tritón X100 0.1%), left 30 min in ice with vortex every 5 min and centrifuged at 10000 rpm for 20 min at 4 °C. Cytoplasm fraction and supernatant were concentrated with TCA. Nuclear fractions with 100 μl de PBS were sonicated employing >20 kHz and testing different intervals of time: pulses (1–5) seconds (30s, 60s).

### Protein purification with nickel–nitrilotriacetic acid (Ni–NTA) metalaffinity chromatography matrices

The Ni–NTA Spin Kit (QIAGEN) was used for purification of the target proteins. The purification was carried out according to the manufacturer’s instructions. We tested to purify either under native or denaturing conditions, with or without the addition of protease inhibitors such as PMSF. Wash buffer of different stringent conditions were also tested (15 mM, 20 mM or 30 mM imidazole) and different elution conditions: pH (acidic values pH 4.5–5.9) or imidazole (100–500 mM).

### Immunization and evaluation of the humoral immune response

The animal experiments were approved by our Institutional Experimentation Animal Committee (CICUAE-INTA). Animal handling and experimental procedures were strictly in accordance with the recommendations in the Guide for the Care and Use of Laboratory Animals of the National Institutes of Health. Maximum efforts were made to minimize mice suffering. Two female BALB/c mice (6–8 weeks old) were used for vaccination. Animals received, by the intra-peritoneal (ip) route, one dose of an oil-based vaccine consisting of 500 ng rSN1 formulated with complete Freund adjuvant, at day 23 another dose with rSN1 emulsed with incomplete Freund adjuvant (IFA) and at days 40 a third dose with extract nuclei expressing rSN1 and IFA. At 30 days 1% heparine blood sample were obtained and at 53 days 1% heparine total blood was collected. Isoflurane was used as anesthetic to collect total mice blood and then euthanized by cervical dislocation.

### Western blot analysis and antibodies

For the over-time study, 10 microliters (μl) of total cell extract were loaded on a SDS-Polyacrylamide Gel Electrophoresis (SDS-PAGE, 10%) and transferred to a nitrocellulose membrane (GE Healthcare). For fractioning study, 20 microliters (μl) of total cell extract, concentrated supernatant, concentrated cytoplasm or nuclear fraction were loaded on a SDS-PAGE, 13.5% and transferred. For purification study 25 μl of washing (total 600 μl) or elution (total 300 μl) buffer were loaded on 10% SDS-PAGE and transferred. For Western blot analysis of anti-rSN1 sera, 40 μl extract of nuclei of Sf9 cells either expressing rSN1 or not were loaded on a 15% SDS-PAGE and transferred. To determine the dilution at which the antibody can be used in the Western blotting technique, we run the same amounts of the antigen (300 μg of purified rSN1 or 30 mg of total crude leaf potato protein) on electrophoresis (SDS-PAGE, 13.5%). Serial dilutions at third were made from total polyclonal serum. Antibody titer was expressed as the inverse of the greatest serial dilution that still gives a positive result. For plant assays, 20 mg of total protein were loaded on 13.5% SDS-PAGE. *Nicotiana benthamiana* leaves were infiltrated with *Agrobacterium tumefaciens* GV3101 carrying SN1ΔSP-Egfp as previously described by Nahirñak et al. [10] and stable potatoes lines were obtained and in vitro maintained by our group. Reversible Ponceau staining as a loading control in Western blots was employed in all membranes. Different antibodies were used to reveal: commercial anti-HIS antibody (1:1000, Amersham Pharmacia Biotech), commercial rabbit anti-GFP (1∶500, Molecular Probes, A6455), serum sample (1:100) and total polyclonal serum (1:100). The membranes were probed with the described antibodies followed by a 1:5000 dilution of goat Anti-Mouse IgG-Alkaline Phosphatase antibody (Sigma-Aldrich). The membrane was developed with the BCIP/NBT Phosphatase Substrate System (Biorad). Molecular weight standards: BenchMark Pre-Stained Protein Ladder or PageRuler Prestained Protein Ladder (Thermo Scientific) were employed.

## Additional files

Additional file 1: Figure S1. Determination of the antibody titer by immunoblots assays. Purified rSN1 (300 μg per lane) or total potato leaf protein extract (30 mg per lane) were employed as epitope. Serial dilutions at third were made from total polyclonal serum: Lane 1: 1/100 dilution; Lane 2: 1/300 dilution; Lane 3: 1/900 dilution; Lane 4: 1/2700 dilution; Lane 5: 1/8100 dilution; Lane 6: 1/24300 dilution; Lane 7: 1/72900 dilution; Lane 8: 1/218700 dilution. (TIFF 361 kb)

Additional file 2: Figure S2. Detection of StSN1 expressed in overexpressing transgenic potato transformed plants. Leaf samples of transgenic and control plants were employed. NT: non-transgenic plant. S1, S3 and S5: StSN1-overexpressing lines. M: BenchMark Pre-Stained Protein Ladder. Western blots analysis revealed with the polyclonal serum anti-rSN1 are shown. Equal amounts of total protein were loaded on 13.5% SDS-PAGE. Intensity of bands was compare using ImageJ software (http://rsb.info.nih.gov/ij/index.html). (TIFF 157 kb)

## Abbreviations

a.k.a.: also known as
AMPs: Antimicrobial peptides
DPI: Days post infection
GASA: Giberellic Acid Stimulated in Arabidopsis
GFP: Green fluorescent protein
GSL1: Gibberellin stimulated-like 1
GST: Glutathione S-transferase
IB: Inclusion bodies
MOI: Multiplicity of infection
PMSF: Phenylmethylsulfonyl fluoride
rSN1: recombinant StSN1
s: seconds
SN2: Snakin-2
SP: Signal peptide
StSN1: *Solanum tuberosum* Snakin-1
