# Supplementary figures and images for: Successful production of the potato antimicrobial peptide Snakin-1 in baculovirus-infected insect cells and development of specific antibodies
Source: BMC Biotechnol. 2017 Nov 9;17:75. doi: 10.1186/s12896-017-0401-2 (PMC5679188; doi:10.1186/s12896-017-0401-2)

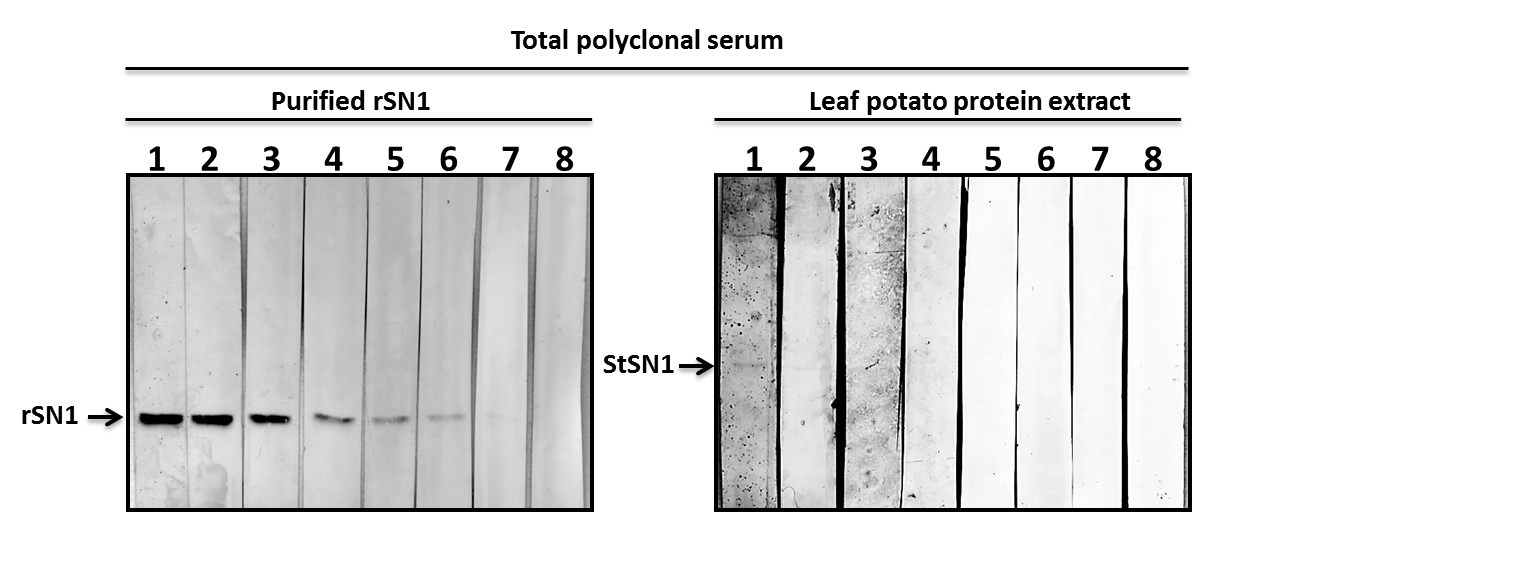

Supplement: Supplementary file 1 — Determination of the antibody titer by immunoblots assays. Purified rSN1 (300 μg per lane) or total potato leaf protein extract (30 mg per lane) were employed as epitope. Serial dilutions at third were made from total polyclonal serum: Lane 1: 1/100 dilution; Lane 2: 1/300 dilution; Lane 3: 1/900 dilution; Lane 4: 1/2700 dilution; Lane 5: 1/8100 dilution; Lane 6: 1/24300 dilution; Lane 7: 1/72900 dilution; Lane 8: 1/218700 dilution. (TIFF 361 kb) [file 12896_2017_401_MOESM1_ESM.tif]

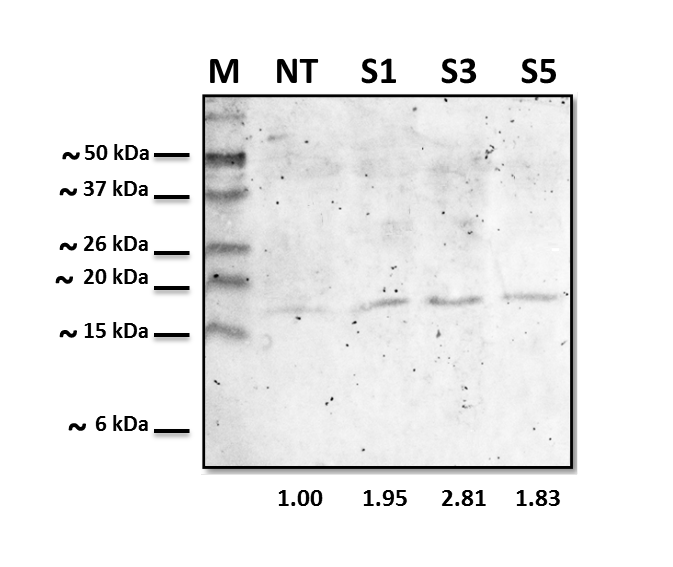

Supplement: Supplementary file 2 — Detection of StSN1 expressed in overexpressing transgenic potato transformed plants. Leaf samples of transgenic and control plants were employed. NT: non-transgenic plant. S1, S3 and S5: StSN1-overexpressing lines. M: BenchMark Pre-Stained Protein Ladder. Western blots analysis revealed with the polyclonal serum anti-rSN1 are shown. Equal amounts of total protein were loaded on 13.5% SDS-PAGE. Intensity of bands was compare using ImageJ software (http://rsb.info.nih.gov/ij/index.html). (TIFF 157 kb) [file 12896_2017_401_MOESM2_ESM.tif]
